# Validating left atrial fractionation and low-voltage substrate during atrial fibrillation and sinus rhythm—A high-density mapping study in persistent atrial fibrillation

**DOI:** 10.3389/fcvm.2022.1000027

**Published:** 2022-10-18

**Authors:** Taiyuan Huang, Juan Chen, Björn Müller-Edenborn, Louisa Mayer, Martin Eichenlaub, Zoraida Moreno Weidmann, Juergen Allgeier, Marius Bohnen, Heiko Lehrmann, Dietmar Trenk, Simon Schoechlin, Dirk Westermann, Thomas Arentz, Amir Jadidi

**Affiliations:** ^1^Department of Cardiology, Arrhythmia Division, Faculty of Medicine, University Heart Center Freiburg-Bad Krozingen, University of Freiburg, Freiburg im Breisgau, Germany; ^2^Department of Cardiology, Affiliated Drum Tower Hospital, Medical School of Nanjing University, Nanjing, China; ^3^Arrhythmia Unit, Department of Cardiology, Hospital Universitario Sant Pau, Barcelona, Spain

**Keywords:** persistent atrial fibrillation, mapping, catheter ablation, prolonged fractionated potential, pulmonary vein isolation, atrial late potentials, low voltage, substrate

## Abstract

**Background:**

Low-voltage-substrate (LVS)-guided ablation for persistent atrial fibrillation (AF) has been described either in sinus rhythm (SR) or AF. Prolonged fractionated potentials (PFPs) may represent arrhythmogenic slow conduction substrate and potentially co-localize with LVS. We assess the spatial correlation of PFP identified in AF (PFP-AF) to those mapped in SR (PFP-SR). We further report the relationship between LVS and PFPs when mapped in AF or SR.

**Materials and methods:**

Thirty-eight patients with ablation naïve persistent AF underwent left atrial (LA) high—density mapping in AF and SR prior to catheter ablation. Areas presenting PFP-AF and PFP-SR were annotated during mapping on the LA geometry. Low-voltage areas (LVA) were quantified using a bipolar threshold of 0.5 mV during both AF and SR mapping. Concordance of fractionated potentials (CFP) (defined as the presence of PFPs in both rhythms within a radius of 6 mm) was quantified. Spatial distribution and correlation of PFP and CFP with LVA were assessed. The predictors for CFP were determined.

**Results:**

PFPs displayed low voltages both during AF (median 0.30 mV (Q1–Q3: 0.20–0.50 mV) and SR (median 0.35 mV (Q1–Q3: 0.20–0.56 mV). The duration of PFP-SR was measured at 61 ms (Q1–Q3: 51–76 ms). During SR, most PFP-SRs (89.4 and 97.2%) were located within LVA (<0.5 mV and <1.0 mV, respectively). Areas presenting PFP occurred more frequently in AF than in SR (median: 9.5 vs. 8.0, *p* = 0.005). Both PFP-AF and PFP-SR were predominantly located at anterior LA (>40%), followed by posterior LA (>20%) and septal LA (>15%). The extent of LVA < 0.5 mV was more extensive in AF (median: 25.2% of LA surface, Q1–Q3:16.6–50.5%) than in SR (median: 12.3%, Q1–Q3: 4.7–29.4%, *p* = 0.001). CFP in both rhythms occurred in 80% of PFP-SR and 59% of PFP-AF (*p* = 0.008). Notably, CFP was positively correlated to the extent of LVA in SR (*p* = 0.004), but not with LVA in AF (*p* = 0.226). Additionally, the extent of LVA < 0.5 mV in SR was the only significant predictor for CFP, with an optimal threshold of 16% predicting high (>80%) fractionation concordance in AF and SR.

**Conclusion:**

Substrate mapping in SR vs. AF reveals smaller areas of low voltage and fewer sites with PFP. PFP-SR are located within low-voltage areas in SR. There is a high degree of spatial agreement (80%) between PFP-AF and PFP-SR in patients with moderate LVA in SR (>16% of LA surface). These findings should be considered when substrate-based ablation strategies are applied in patients with the left atrial low-voltage substrate with recurrent persistent AF.

## Introduction

Pulmonary veins (PVs), as a major trigger for AF, are considered the primary target site during ablation (1). While high rates (75–85%) of sinus rhythm maintenance are achieved with the single pulmonary vein isolation (PVI) approach in both paroxysmal and persistent AF patients without left atrial low-voltage substrate, this approach results in reduced (45–55%) success rates in those presenting significant low-voltage substrate in the LA (2, 3). Previous randomized controlled trials (RCTs) failed to demonstrate significant superiority of empiric extensive ablation strategies over PVI-alone in patients with persistent AF (4–6). However, termination of AF by local ablation within the left atrium before performing PVI further indicated the existence of trigger sites outside of PVs (7, 8). In the last years, two similar substrate-based approaches were reported for ablation of persistent AF, resulting in improved arrhythmia freedom rates of 70% at 1-year follow-up in persistent AF patients who present significant left atrial low-voltage substrate: (1) PVI plus mapping and ablation of low voltage and prolonged fractionated potentials (PFP) during AF (PFP-AF) (8–10). (2) PVI plus ablation of low voltage and fractionated potentials in SR (PFP-SR): the electrograms are characterized by prolonged deflections following a first atrial depolarization and were reported by our team and Yang et al. (11) and named prolonged fractionated potential in SR (PFP-SR) or atrial late potentials (ALP) (12). While PFP-AF and PFP-SR are observed/mapped during different rhythms, their spatial correlation and distribution remain unknown. The current study aims to analyze the spatial distribution, and quantify and identify the predictors of concordance between PFP-AF and PFP-SR. In addition, the relationship of PFPs to the underlying low-voltage substrate is assessed.

## Materials and methods

### Study population

The study cohort consists of 38 consecutive patients presenting for their first catheter ablation procedure for symptomatic persistent AF (lasting for > 7 days and < 12 months). Exclusion criteria were the presence of atrial thrombus on pre-procedural transesophageal echocardiography or previous atrial ablation or surgery. The study protocol followed the principles of the Helsinki Declaration and was approved by the Human Research Ethics Committee of the University of Freiburg. All patients provided written informed consent to participate in this study.

### High-density voltage mapping in both atrial fibrillation and sinus rhythm

Left atrial mapping was performed under general anesthesia *via* transseptal access with more than 1,200 sites per patient during both AF and SR. Maps were acquired using a 20-pole circumferential catheter (Lasso-Nav, diameter 15–25 mm; electrode size: 1 mm; electrode spacing: 2–6–2 mm; Biosense Webster) in combination with the Carto 3 (V7) electro-anatomical mapping system. The electro-anatomic maps were acquired after respiratory gating with a low-interpolation setting (Carto 3 system, 18–20 for LA geometry acquisition). The interior projection distance (“filtering”) was set at a distance of 5 mm to the surface of LA geometry. Areas demonstrating low voltage when mapped with the 20-pole lasso catheter were reconfirmed using a contact force-enabled mapping catheter with a contact threshold of > 3 g. After mapping during the initial rhythm of AF or SR, individuals underwent electrical cardioversion to SR or induction to AF by rapid pacing to continue mapping in different rhythms. Only mapping sites that were within an adjacent distance of 5 mm contributed to voltage maps.

For voltage mapping during AF, we ensured a high mapping density (>1,000 points per atrium and rhythm). The window of interest was set to the average AF cycle length, to enable measurement of the peak-to-peak amplitude of a single AF beat (because the current CARTO system does not allow measurement of average voltage of multiple consecutive beats). However, because of the high-mapping density, multiple mapping points are recorded within a small area (<0.3 cm^2^), allowing to calculate and display the average voltage of these mapping points that project to the same area. The recorded high-density voltage maps in AF are therefore highly consistent with regard to the extent and distribution of low-voltage areas (13).

Left atrial area (LAA) and left atrial volume (LAV) were measured using the CARTO mapping system with exclusion of the PV ostia and mitral annulus.

### Definition of prolonged fractionated potentials-in-atrial fibrillation and prolonged fractionated potentials-in-sinus rhythm

**FIGURE 1:**
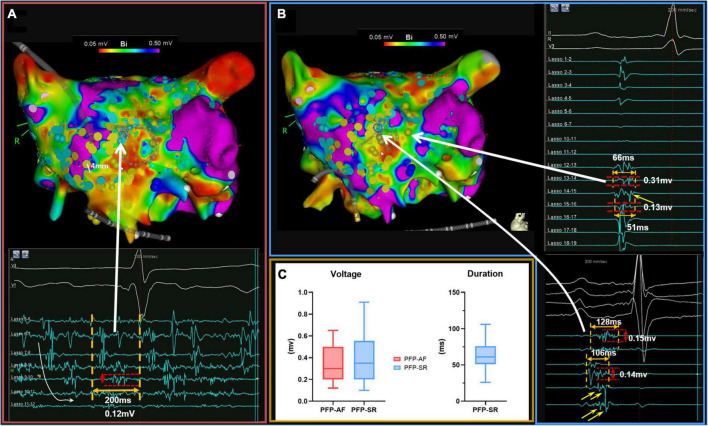
Illustration of PFP-AF and PFP-SR. **(A,B)** Illustrate voltage mapping during AF and SR, respectively. A bipolar threshold of 0.5 mV was adopted in both maps to identify LVA. Mapping points presenting PFP-AF and PFP-SR are annotated in light blue and yellow, respectively. Panel **(A)** illustrates electrogram examples of PFP-AF and panel **(B)** examples of PFP-SR. PFP-AF is defined as electrograms with prolonged activity lasting ≥70% local AF cycle length (Lasso 8–9 and 9–10) on a single or on multiple consecutive bipoles, with high-temporal consistency (8/10 consecutive AF beats with an electrogram “observation period” of 15 s at the same location. The curved white arrow illustrates spatio-temporal dispersion on Lasso 5–6 to Lasso 10–11). PFP-SR is defined as electrograms with ≥5 deflections or electrograms with a delayed electrogram component following a first depolarization event on a single bipolar recording in SR. Characteristic delayed electrogram components of PFP-SR are illustrated with yellow arrows in panel **(B)**. **(C)** Depicted the electrophysiological characteristics of PFP-AF (red) and PFP-SR (blue). Voltage and duration are expressed as median, 25th and 75th percentiles. PFP, prolonged fractionated potential; AF, atrial fibrillation; SR, sinus rhythm.

**FIGURE 2:**
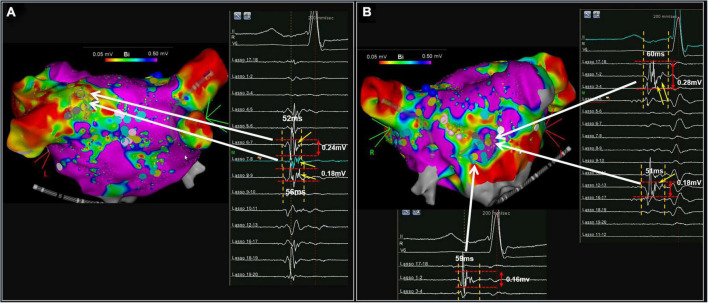
Illustrates another patient example with PFP-SR [posterior view in **(A)** and anterior view in the **(B)**]. Characteristic delayed electrogram components of PFP-SR are illustrated with yellow arrows.

Illustrations of PFP-AF and PFP-SR are presented in Figures 1A,B, 2A,B, respectively. As reported previously (9, 12), PFP-AF was defined as electrograms lasting ≥70% local AF cycle length (AFCL) on single or multiple neighboring bipoles of the circumferential catheter, with high-temporal consistency (8/10 consecutive AF beats with an electrogram “observation period” of 15 s at the same location; Figure 1A). PFP-SR were defined as electrograms with ≥5 deflections or electrograms with a delayed low-voltage component (median voltage 0.35 mV) following a first component (depolarization event) on a single bipolar recording in SR (see Figures 1B, 2A,B). The left atrium was divided into six segments, including the anterior wall, posterior wall, septum, roof, inferior, wall, and lateral wall (see Supplementary Figure 1). Areas presenting PFP in each map (AF or SR) were annotated on the LA geometry during LA mapping. The number of areas presenting PFP-AF or PFP-SR was calculated within a radius of 6 mm covering neighboring mapping sites in each segment of the left atrium.

### Definition of the low-voltage area in both atrial fibrillation and sinus rhythm

In the current study, quantification of the low-voltage substrate in each rhythm was performed separately using a bipolar threshold of 0.5 mV in both AF and SR mapping. Low-voltage area (LVA) was therefore reported as separate results in respective rhythm and the percentage of LVA was calculated by the absolute LVA divided by the total surface of LA excluding mitral annulus and PV antra. The percentage of PFP-SR located within low-voltage areas (in SR) < 0.5 mV as well as LVA < 1.0 mV was assessed.

### Definition of concordance of fractionated potentials during atrial fibrillation and sinus rhythm

After annotation of PFP in both rhythms, their spatial correlation to LVA was quantified as the percentage of PFP located in LVA < 0.5 mV (or in 1 cm border of LVA) from the respective map. Subsequently, we overlapped SR and AF maps to visualize the spatial distribution between PFP-AF and PFP-SR.

Although the electro-anatomical system in CARTO has high-spatial accuracy (<1 mm), the same atrial location may be slightly shifted/moved between the two rhythms (slight spatial discrepancies between the first map in AF and the second map in SR). This may be due to different factors: (1) slight movement of the patient during the electrical cardioversion; (2) change in the left atrial volume and contraction behavior during AF vs. SR. To account for these slight location differences between the AF and SR maps, we therefore defined “concordant fractionated potentials (CFP; Figures 1A,B),” if concomitant PFP occurred at LA locations within a 6 mm radius during both SR and AF.

## Statistical analysis

Continuous variables were expressed as mean ± SD or median ± interquartile range based upon distribution status, categorical variables were expressed as frequency and percentage (%). Comparison between two paired groups was performed using the paired *t*-test or the Wilcoxon rank test. Categorical variables were expressed as frequency and percentage (%) and were compared by the Chi-square test or Fisher’s exact test. Correlation between variables was performed using Pearson or Spearman correlation analyses. In the current study, the percentage of CFP was defined as an independent variable. As it was defined as the proportion of CFP in PFP-AF or PFP-SR. The absolute number of PFP in either map was hereby considered as an intermediate factor. Additionally, we used the percentage of LVA as a parameter to quantify the extent of LVA, which was divided by the surface of LA. In this context, LA surface was also classified as an intermediate factor. After the exclusion of potential intermediate factors, linear regression was performed using a stepwise approach to identify significant predictors of CFP. A reference map (SR or AF map) was selected based on results from linear regression. According to the data distribution of CFP percentage from the reference map, the cut-off value (median or mean value) between high and low concordance in PFP was determined. The receiver operating characteristics (ROC) curve was performed to estimate the discriminative power of predictors to identify the extent of concordance (high or low) and to determine the optimal threshold of LVA extent by the Youden index. A radar chart was generated to visualize the diagnostic performance of the threshold regarding sensitivity, specificity, positive predictive value, negative, predictive value, and accuracy. Statistical analysis was performed with SPSS version 27.0 for Macintosh (IBM-Corporation, Armonk, NY), and GraphPadPrism-V9.0 for Macintosh (GraphPad Software, LaJolla, CA, USA). A radar chart was performed using the ggradar package of R version 4.2.1.

## Results

### Patient characteristics

As listed in Table 1, the average age of participants was 63.76 ± 10.32 years, and 57.9% were female. Hypertension and diabetes were prevalent in 55.3 and 13.2% of study participants, respectively. Twenty-eight participants (73.7%) presented initially with AF and underwent electrical cardioversion after voltage mapping in AF was completed. Six patients experienced reinitiation of AF shortly (within 20 s) after electrical cardioversion to SR. In these cases, AF voltage mapping was first acquired. After completion of PVI, all six patients were cardioverted to SR and maintained SR, allowing them to acquire an LA voltage map in SR. Ten participants (26.3%) were initially in SR and underwent AF induction after voltage mapping in SR.

**TABLE 1:** Baseline characteristics of the study group (*n* = 38).

Variable	Value
Age, yrs	63.76 ± 10.32
Female, *n* (%)	22 (57.9%)
BMI, kg/m^2^	26.80 ± 7.65
LAD, mm	42.35 ± 6.95
LA surface, cm^2^	106.3 ± 37.65
LAV, ml	156.51 ± 39.21
LVEDD, mm	48.78 ± 5.94
LVESD, mm	32.51 ± 6.79
LVEF,mm	56.35 ± 11.33
Hypertension, *n* (%)	21 (55.3%)
Diabetes, *n* (%)	5 (13.2%)
CAD, *n* (%)	6 (15.8%)
Stroke/TIA, n (%)	1 (2.6%)
CHA2DS2-Vasc Score	2.00 ± 1.00
Creatine, (mg/dl) Mapping points	73.96 ± 17.52 1,258 ± 524

BMI, body mass index; LAD, left atrial diameter; LA, left atrial; LAV, left atrial volume; LVEDD, left ventricular atrial end-diastolic diameter; LVESD, left ventricular atrial end-systolic diameter; LVEF, left ventricular ejection fraction; CAD, coronary artery disease; TIA, transient ischemic attack.

### Electrophysiological characteristics and spatial distribution of prolonged fractionated potentials in atrial fibrillation and sinus rhythm maps

In Figures 1A,B, 2A,B, examples of PFPs are provided in both AF and SR. The distribution of PFP-SR and PFP-AF is illustrated as yellow and blue markers, respectively, that are projected to both maps (voltage in AF in Figure 1A and voltage in SR in Figure 1B). The late components of PFP-SR and the PFP-AF sites display low voltages: a median voltage of 0.30 mV (Q1–Q3: 0.20–0.50 mv) in PFP-AF and 0.35 mV (Q1–Q3: 0.20–0.56 mv) in PFP-SR, and the median duration of PFP-SR measured 61 ms (Q1–Q3: 51–76 ms; Figure 1C).

### Spatial distribution of prolonged fractionated potentials during sinus rhythm and atrial fibrillation

**FIGURE 3:**
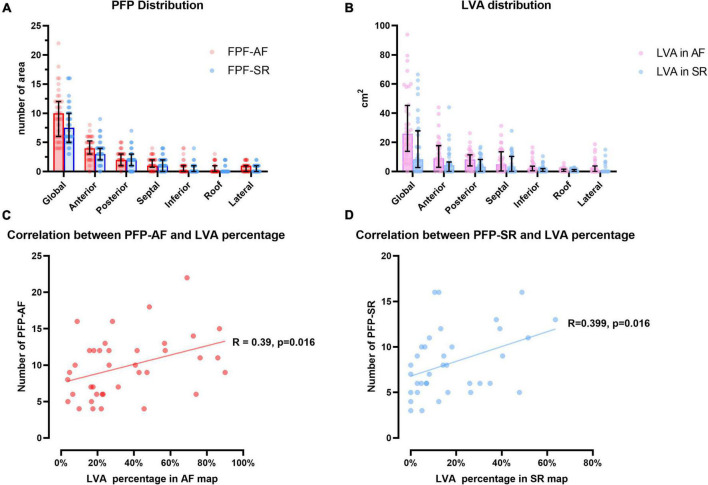
Distribution of areas presenting prolonged fractionated potential and low voltage area. **(A)** Illustrates the number of areas presenting PFP-AF (red) and PFP-AF (blue) in global LA and each segment. **(B)** Illustrates the distribution LVA in LA at bipolar threshold of 0.5mV in AF (violet) and SR (light blue) maps. All values are expressed as median (upper margin of bar plot), 25th and 75th percentile (error bar in black). **(C,D)** Display the correlation between PFP and percentage of LVA during AF mapping (red) and SR mapping (blue), correlation coefficient and p value are annotated, respectively. PFP, prolonged fractionated potential; AF, atrial fibrillation; SR, sinus rhythm; LVA, low voltage area; LA, left atrium.

The number of areas presenting PFP-AF was significantly higher than those presenting PFP-SR (median: 9.5, Q1–Q3: 6.0–12.0 in PFP-AF vs. median: 8.0, Q1–Q3: 5.75–10.25 in PFP-SR, *p* = 0.005). Figure 3A and Table 2 depict the number and percentage of areas manifesting PFP-AF and PFP-SR in each segment as well as the global LA, respectively. The anterior wall (both in AF and SR) demonstrated the most frequently PFP among all segments with a comparable proportion between PFP-AF and PFP-SR (PFP-AF: 42.3%, Q1–Q3: 33.3–50.0% vs. PFP-SR: 40.0%, Q1–Q3: 33.3–51.6%, *p* = 0.531). The posterior wall and septum demonstrated more than 20 and 15% of total PFP areas in both rhythms without a significant difference between PFP-AF and PFP-SR (*p* = 0.266 in the posterior wall, *p* = 0.088 in the septum). The remaining segments, including the roof, inferior wall, and lateral wall, on the other hand, showed significantly less prevalence of PFP-AF and PFP-SR.

**TABLE 2:** Spatial distribution of prolonged fractionated potentials-atrial fibrillation (PFP-AF) and prolonged fractionated potentials-sinus rhythm (PFP-SR).

	PFP-AF			PFP-SR			*p* value
Number of areas	Median	Q1	Q3	Median	Q1	Q3	
Global LA	9.50	6.00	12.00	8.00	5.75	10.25	0.005
Anterior wall	4.00	2.75	5.25	3.50	2.00	4.00	0.059
Posterior wall	2.00	1.75	3.00	2.00	1.00	3.00	0.023
Septum	1.00	0.75	2.00	1.00	1.00	2.00	0.661
Roof	0.00	0.00	1.00	0.00	0.00	0.00	0.011
Inferior wall	0.00	0.00	1.00	0.00	0.00	1.00	0.088
Lateral wall	1.00	0.00	1.00	0.00	0.00	1.00	0.046

	**PFP-AF**			**PFP-SR**			***p* value**
**Distribution (%)**	**Median**	**Q1**	**Q3**	**Median**	**Q1**	**Q3**	

Anterior wall	42.26	33.33	50.00	40.00	33.33	51.56	0.531
Posterior wall	21.83	16.67	33.33	22.65	9.58	34.09	0.266
Septum	15.48	4.17	20.00	17.42	7.81	25.89	0.088
Roof	0.00	0.00	10.28	0.00	0.00	0.00	0.038
Inferior wall	0.00	0.00	11.11	0.00	0.00	10.28	0.747
Lateral wall	6.46	0.00	11.11	0.00	0.00	11.46	0.751

PFP, prolonged fractionated potentials; AF, atrial fibrillation; SR, sinus rhythm; LA, left atrium. Q1, 25th percentile; Q3, 75th percentile.

### Spatial distribution of low voltage areas and prolonged fractionated potentials in atrial fibrillation and sinus rhythm

As presented in Figure 3B, LVA in AF and SR maps were separately quantified using a bipolar voltage threshold of 0.5 mV in each rhythm. During AF, the absolute extent of LVA was measured at 25.85 cm^2^ (Q1–Q3: 13.78–45.28 cm^2^) and in SR at 10.45 cm^2^ (Q1–Q3: 4.08–32.88 cm^2^). The relative extent of LVA (defined as the percentage of LA surface displaying LVA) was significantly more extensive in AF (median: 25.2%, Q1–Q3:16.6–50.5%) than in SR (median: 12.3%, Q1–Q3: 4.7–29.4%, *p* = 0.001). Detailed LVA distribution and comparison between AF and SR maps in each segment are listed in Supplementary Table 1. Notably, among all sites presenting PFP, 100% (Q1–Q3: 89.7–100%) of PFP-AF were located in (or in the 1 cm-border area of) LVA (<0.5 mV in AF) with a significant correlation between the number of PFP-AF and the extent of LVA in LA (Correlation coefficient: 0.39, *p* = 0.016; Figure 3C). During SR mapping, 89.4% (Q1–Q3: 72.1–100%) of PFP-SR located in (or within the 1 cm bordering area of) LVA (<0.5 mV in SR). Moreover, most PFP-SRs (97.2%) are located within LVA (<1.0 mV in SR). Accordingly, a significant correlation between PFP-SR and the extent of LVA (<0.5 mV in SR) during SR was observed (Correlation coefficient: 0.399, *p* = 0.016; Figure 3D).

### Evaluation of concordance of fractionated potentials

**FIGURE 4:**
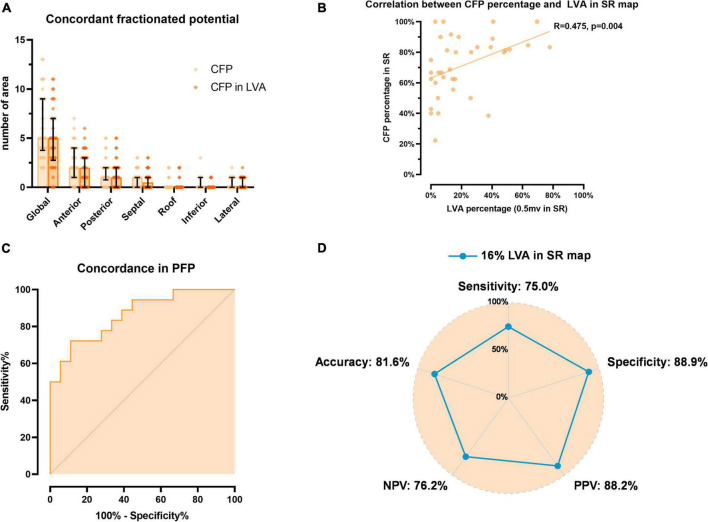
Evaluation of concordance of fractionated potentials (CFP). **(A)** Illustrates the distribution of areas presenting CFP and those located in LVA in SR map. **(B)** Displays the correlation between percentage of CFP in PFP-SR and LVA extent in SR map. **(C)** Displays the ROC curve of high concordance (>80%) between PFP-AF and PFP-SR, which is predicted by LVA extent in SR. **(D)** Illustrates the diagnostic performance of optimal LVA extent threshold (16% of LA displaying LVA) to predict high concordance of fractionated potentials. CFP, concordance of fractionated potentials; LVA, low voltage area; PFP, prolonged fractionated potential; SR, sinus rhythm; AF, atrial fibrillation; LA, left atrium; PPV, positive predictive value; NPV, negative predictive value.

We defined “CFP (Figures 1A,B)” if concomitant prolonged fractionation occurred at LA locations within a 6 mm radius during both SR and AF. The number of areas with CFP in the SR map is illustrated in Figure 4A, in accordance with PFP distribution, CFP also displayed preponderant distribution in the anterior, posterior wall, and septum. Moreover, we compared the percentage of CFP between PFP-AF and PFP-SR: CFP was found at a significantly higher proportion of PFP-SR sites than sites displaying PFP-AF (in PFP-SR: 80%, Q1–Q3: 61.88–89.17% vs. in PFP-AF: 58.6% CFP, Q1–Q3: 48.86–81.39%, *p* = 0.008). Subgroup analysis in each segment is listed in Table 3; however, a significant difference in CFP percentage was only observed in the anterior wall (*p* = 0.008).

**TABLE 3:** Percentage of concordance of fractionated potentials (CFP) in prolonged fractionated potentials-atrial fibrillation (PFP-AF) and prolonged fractionated potentials-sinus rhythm (PFP-SR).

	CFP as percentage of PFP-AF			CFP as percentage of PFP-SR			*P* value
Distribution (%)	Median	Q1	Q3	Median	Q1	Q3	
Anterior wall	66.67	47.50	87.50	81.67	50.00	100.00	0.008
Posterior wall	66.67	25.00	100.00	90.00	25.00	100.00	0.452
Septum	50.00	0.00	100.00	50.00	0.00	100.00	0.504
Roof	0.00	0.00	50.00	0.00	0.00	56.25	0.414
Inferior wall	0.00	0.00	0.00	0.00	0.00	0.00	0.18
Lateral wall	0.00	0.00	100.00	0.00	0.00	100.00	0.102

CFP, concordance of fractionated potentials; PFP, prolonged fractionated potential; AF, atrial fibrillation; SR, sinus rhythm; Q1, 25th percentile; Q3, 75th percentile.

### Correlation between concordance of fractionated potentials and low voltage areas

Given the significantly higher concordance between PFP-AF and PFP-SR that was observed during SR mapping, the correlation between CFP and LVA was first analyzed in the SR voltage map. The distribution of CFP in LVA is shown in Figure 4A. In accordance with PFP in LVA, CFP in LVA displayed a consistent pattern in spatial distribution that mostly in the anterior wall, followed by the posterior wall and septum. Notably, a significant positive correlation was also observed between the percentage of CFP in PFP-SR and LVA extent in SR (Correlation coefficient: 0.475, *p* = 0.004; Figure 4B). This finding reveals that in patients with significant underlying LVA in SR, the percentage of concordant fractionation is high, whereas patients with no/little underlying LVA in SR display a highly variable degree of concordance in fractionation between both rhythms, which may be related to functional and rhythm- and rate-dependent fractionation.

Additionally, we performed a sensitivity analysis to test the correlation between LVA in AF and CFP: By setting the AF map as a reference, however, the correlation between CFP percentage in PFP-AF and LVA extent in the AF map failed to reach significance (Correlation coefficient: 0.201, *p* = 0.226; Supplementary Figure 2).

### Predictor of concordance of fractionated potentials by linear regression

In the current study, the percentage of CFP was defined as an independent variable. As it was defined as the proportion of CFP in PFP-AF or PFP-SR. The absolute number of PFP in either map was hereby considered as an intermediate factor. Additionally, we used the percentage of LVA as a parameter to quantify the extent of LVA, which was divided by the surface of LA. In this context, LA surface and the number of PFP-AF or PFP-SR were excluded before linear regression for predictors of CFP percentage. As listed in Table 4, the extent of LVA in SR was the only significant predictor for percentage of CFP in PFP-SR (*p* = 0.009). However, when using CFP percentage in PFP-AF as an independent variable, no significant predictor was identified, which is consistent with the results in correlation analysis. A sensitivity analysis was performed to validate the predictive value of LVA for CFP by setting the absolute number of areas presenting CFP as an independent variable. In this context, LVA in AF and SR maps were analyzed as an absolute value, and the LA surface was considered as a confounder instead. The number of PFP-AF and PFP-SR remained intermediate factors. As a result (Supplementary Table 2), LVA in SR was the only significant predictor for the number of CFP (*p* = 0.004), whereas the LVA in AF remained insignificant (*p* = 0.128).

**TABLE 4:** Stepwise linear regression for a predictor of concordance of fractionated potentials.

	β coefficient	*p* value	Collinearity statistics	
Selected variable			Tolerance	VIF
LVA percentage in SR map	0.393	0.009	1	1
Excluded variable				
Age	0.066	0.687	0.924	1.082
Sex	–0.036	0.82	0.998	1.002
Intial Rhythm	0.015	0.927	0.918	1.09
BMI	–0.088	0.583	0.963	1.038
LAD	–0.084	0.615	0.89	1.123
LAV	0.01	0.952	0.899	1.113
LVEF	–1.41	0.372	0.991	1.009
Creatine	0.014	0.929	0.946	1.057
Diabets	–0.08	0.617	0.975	1.026
Stroke	0.203	0.196	0.996	1.004
Hypertension	–0.227	0.155	0.953	1.05
CAD	0.135	0.394	0.984	1.016

LVA, low-voltage area; SR, sinus rhythm; VIF, variance inflation factor, BMI, body mass index; LAD, left atrial diameter; LAV, left atrial volume; LVEF, left ventricular ejection fraction; CAD, coronary artery disease.

### The extent of low voltage areas in sinus rhythm predicts the degree of concordance in fractionation

Based on the aforementioned results, we used the SR map as a reference and dichotomized the CFP percentage in SR to classify “high concordance” and “normal concordance.” Based on data distribution (median: 80%, Q1–Q3: 61.88–89.17%), we defined a CFP percentage of 80% as a cut-off value between high concordance and normal concordance groups. Subsequently, the optimal threshold of LVA percentage in the SR map was determined using ROC analysis. As shown in Figure 4C, an AUC value of 0.864 (95%CI: 0.747–0.981) was achieved. After calculating the Youden index, the optimal threshold was determined as 16% of LVA percentage in the SR map with a sensitivity of 75% and specificity of 88.9%, the diagnostic performance of this threshold was calculated using a confusion matrix and illustrated in Figure 4D.

## Discussion

The current study revealed four main findings: 1. PFPs are more frequent and low-voltage areas more extensive when mapping is performed during AF than SR. 2. PFPs both in AF and SR maps predominantly locate to the anterior, posterior wall, and septal LA walls. 3. Compared to prolonged fractionated sites in AF, those identified during sinus rhythm (PFP-SR) more frequently (in 80%) showed concomitant/concordant fractionation during both rhythms. 4. Presence of ≥16% low-voltage extent (with mapping in SR and <0.5 mV threshold) was highly predictive of a high degree of spatial concordance (above 80%) between PFP-AF and PFP-SR.

### Electrogram fractionation and arrhythmogenesis

Electrogram fractionation may result from wave collision, pivoting, slow conduction, or conduction block (14). Substrate-guided ablation therapy for persistent AF may be necessary for a subset of patients who do not respond well to PVI and who do present atrial low-voltage substrate harboring fractionated delayed potentials. However, selective identification and ablation of fractionated sites that are involved in arrhythmogenesis and slow conduction are necessary to limit harm to healthy atrial myocardium. We recently identified PFP-SR, also named “atrial late potentials (ALP),” as a potential underlying slow conduction substrate located at and around acute AF termination sites (12). Moreover, Yang et al. reported that a strategy of PVI plus ablation of PFP-SR and low-voltage areas mapped in SR improved outcomes in persistent AF patients (11). In a similar study, we reported improved sinus rhythm rates by performing PVI plus selective ablation of low-voltage areas with selective PFP-AF that extended > 70% of the local AF cycle length and were observable with high-temporal consistency (≥80% of beats displayed prolonged or rapid activity) (9). However, the relationship of PFPs in SR and AF to each other and to the underlying low-voltage substrate has not been assessed yet.

### Definitions and differences of complex fractionated electrograms, AF-nest, and prolonged fractionated potentials during atrial fibrillation and sinus rhythm

While complex fractionated electrograms in AF (CFAE) refer to a non-specific classification comprising all kinds of fractionated electrograms during AF (without further precision of their duration, frequency, number of deflections, duration or percentage of the continuous activity, or their voltage), we previously defined PFP-AF (12) with following characteristics: “electrograms in AF with high-temporal consistency (≥80% of AF beats) for the following patterns: duration ≥70% of the local AF cycle length (AFCL) on a single or on multiple neighboring bipoles of the circumferential catheter or electrograms with continuous activity [see Figure 1A and (9, 12)]. PFP-SR or ALPs were defined as electrograms with ≥5 deflections (11) or electrograms with a delayed low-voltage component (median voltage 0.35 mV) following a first depolarization event on a single bipolar recording in SR, as illustrated in Figures 1B,C, 2. In contrast, Pachon et al. define “AF-nest” potentials as fractionation without a relationship to voltage values or low-voltage areas (15). Moreover, using an altered filter setting, they convert high-voltage components of bipolar electrograms recorded with 30–500 Hz to low-voltage highly fractionated signals using filter settings of 300–500 Hz. In addition, they focus and rely on the frequency spectrum of bipolar signals (15).

### Rhythm- and substrate-dependency of electrogram fractionation

The current work reveals that prolonged fractionation occurs more frequently during AF than in SR. Similarly, the underlying low-voltage substrate is more extensive in AF than in SR, suggesting that some of the fractionations and low-voltage areas in AF might be functional (e.g., due to rapid propagation in AF, but not due to fixed slow conducting tissue). Moreover, we observed concordant (occurring during both AF and SR) prolonged fractionation with high consistency (>80%) in patients with underlying low-voltage substrate > 16% of LA surface area during SR mapping with <0.5 mV threshold SR (e.g., patient in Figure 2). In contrast, substantial discrepancies between PFP-AF and PFP-SR were observed in patients who had no/little underlying (fixed) low-voltage substrate during SR. Therefore, ablation therapy targeting PFP-AF or LVA in AF will result in larger ablated tissue than strategies targeting PFP-SR or LVA in SR.

### Substrate mapping for the guidance of ablation therapy in persistent atrial fibrillation

The current study revealed that both LVA and prolonged fractionated sites are more extensive and frequent when mapping is performed in AF than SR. However, irrespective of the underlying rhythm (AF or SR), several recent catheter ablation studies revealed that PVI plus additional ablation of low-voltage areas and prolonged fractionated potentials may improve the rate of sinus rhythm maintenance at 12 months in the subset of patients with the low-voltage substrate. Arrhythmia freedom was improved in most studies from 50% to about 70% after 12 months (9, 11, 16, 17). As targeting the fewer ablation targets (identified in SR rather than AF) in SR results in the same success rates (as if the mapping was done in AF), current substrate-based ablation therapies should be reserved for PVI-non-responders who present low-voltage substrate in SR. In these patients, the substrate-guided strategy should primarily map and target low voltage and PFP-sites in SR (instead of AF) to limit harm to the atrial myocardium.

Future studies should assess if PFP-AF sites with high-temporal consistency (if repeated mapping at those sites confirms PFP-AF within a mapping interval of 30 min) have a higher concordance to underlying slow conduction substrate during SR and to PFP-SR, and might therefore represent a potential ablation target.

### Durable PVI as the main ablative treatment strategy in paroxysmal and persistent AF

PVI is the cornerstone of catheter ablation for AF with high-arrhythmia freedom rates at 1 year both in paroxysmal and persistent AF when using the recent ablation technologies with a high rate of durable PVI (3, 18). However, multiple studies have shown high-arrhythmia recurrence rates in patients with left atrial low-voltage substrate (9, 16, 17). We recently reported that left atrial low-voltage substrate is most frequently found in persistent AF patients aged over 65 years and predominantly in women (19). Although initial single-center studies revealed improved arrhythmia freedom rates in patients with the left atrial low-voltage substrate with a “PVI plus ablation of LVA,” the recent randomized prospective study STABLE-SR-II did not report a significant improvement in outcome with additional substrate ablation (3). Although Yang et al. targeted low-voltage areas in SR <0.4 mV and transitional zones <1.3 mV and included ablation of fractionated potentials in SR, this approach did not significantly improve arrhythmia freedom. However, as the authors of that study commented, STABLE-SR-II included 300 persistent AF patients with an average age of 60 years only, most of whom (68%) were men. Therefore, only 41% of patients randomized to “PVI plus low-voltage ablation” actually had significant low-voltage substrate, and the remaining 59% underwent PVI-only because of the absence of low-voltage areas. In STABLE-SR-II, patients presenting low-voltage areas had a low burden of the low-voltage substrate (median low-voltage area: 4.6% of LA surface). These factors might have minimized the differences between the groups. The rate of arrhythmia freedom at 18 months was 56.5% in patients with atrial low-voltage substrate who underwent a PVI-only approach, whereas additional ablation of low-voltage areas achieved a 64.8% success rate. The study was underpowered and could not detect an outcome difference of 8%. Future larger-scaled studies are needed to assess if ablation of low-voltage areas <0.5 mV and elimination of fractionated late potentials may improve outcomes in those AF patients with more extensive low-voltage substrate who have reduced success rates after PVI-only.

Two further clinical studies have assessed the additional value of substrate-based ablation targeting left atrial late Gadolinium-enhanced areas (LGE) in MRI compared to a PVI-only approach. Although different MRI post-processing techniques were used to detect left atrial LGE-areas (DECAAF-II Trial used the Utah method, whereas, in the ALICIA Trial, the image-intensity-ratio > 1.2 method was applied), both studies revealed the absence of additional benefit of LGE-ablation to a PVI-only strategy (20, 21).

Therefore, existing clinical evidence supports a high-quality circumferential PVI as the primary ablation approach in both paroxysmal and persistent AF patients. A potential positive impact of additional low-voltage-based ablation should be evaluated in large prospective clinical studies, which focus on patients with the low-voltage substrate. Low-voltage-based substrate ablation may be considered in those patients who present atrial low-voltage areas and recur after a first PVI procedure. If substrate-based ablation is performed in PVI-non-responders, prolonged fractionated potentials (atrial late potential and low-voltage areas) should be mapped and targeted during sinus rhythm to prevent extensive ablation with harm to non-arrhythmogenic atrial myocardium.

A recent case series by Hirokami et al. revealed that highly fractioned PFP-SR sites (with ≥7 deflections) collocated to non-PV trigger sites in patients with AF recurrence after a previous PVI approach (22). Their results confirm our previous observations that PFP-SR frequently occurs at AF driver sites (as identified by acute termination upon focal ablation) and may constitute the basis for future substrate-based ablation approaches in patients with AF or atrial tachycardia and underlying fibrotic disease (12).

## Limitations

We reported the spatial distribution of PFP in both AF and SR along with the correlation between their concordance and LVA extent. The presented mapping results are based on a limited patient cohort (38 persistent AF patients) who presented for their first mapping and AF ablation procedure. We used a 20-pole Lasso catheter (1 mm electrode size, 2–6–2 mm spacing), which has similar mapping resolution/properties as a PentaRay catheter. Bipolar voltage maps were acquired at high density (mean of 1,250 mapped LA sites per rhythm) at multiple bipole positions during mapping to eliminate inaccuracies due to the angle of bipole to wavefront direction (23, 24).

## Conclusion

Substrate mapping in SR vs. AF reveals smaller areas of low voltage and fewer sites with PFP. PFP-SRs are located within low-voltage areas in SR. There is a high degree of spatial agreement (80%) between PFP-AF and PFP-SR. These findings should be considered when substrate-based ablation strategies are applied in patients with the left atrial low-voltage substrate with recurrent AF despite previous PVI.

## Data availability statement

The raw data supporting the conclusions of this article will be made available by the authors, without undue reservation.

## Ethics statement

The studies involving human participants were reviewed and approved by Ethics Committee of University of Freiburg. The patients/participants provided their written informed consent to participate in this study.

## Author contributions

All authors listed have made a substantial, direct, and intellectual contribution to the work, and approved it for publication.
