# Supplementary material for: Validating left atrial fractionation and low-voltage substrate during atrial fibrillation and sinus rhythm—A high-density mapping study in persistent atrial fibrillation
Source: Front Cardiovasc Med. 2022 Oct 18;9:1000027. doi: 10.3389/fcvm.2022.1000027 (PMC9622778; doi:10.3389/fcvm.2022.1000027)
Supplement: Supplementary file 1 [file Data_Sheet_1.docx]

**Validating Left Atrial Fractionation-Substrate During Atrial Fibrillation and Sinus Rhythm – a High Density Mapping Study in Persistent Atrial Fibrillation**

**Supplemental Appendix**

**Supplemental tables (Page 2 - 3)**

**Supplemental figure (Page 4 - 5)**

|  | LVA (0.5mv in AF) | | | LVA (0.5mv in SR) | | | p value |
| --- | --- | --- | --- | --- | --- | --- | --- |
| Area (cm^2^) | Median | Q1 | Q3 | Median | Q1 | Q3 |  |
| Global LA | 25.85 | 13.78 | 45.28 | 10.45 | 4.08 | 28.00 | <0.001 |
| Anterior wall | 9.49 | 2.98 | 18.46 | 3.80 | 0.00 | 6.97 | <0.001 |
| Posterior wall | 7.62 | 3.23 | 11.58 | 3.15 | 0.00 | 7.01 | <0.001 |
| Septal wall | 6.12 | 0.32 | 12.98 | 0.99 | 0.00 | 9.27 | 0.017 |
| Roof | 0.83 | 0.00 | 1.61 | 0.00 | 0.00 | 1.39 | 0.088 |
| Inferior wall | 1.81 | 0.54 | 4.54 | 0.43 | 0.00 | 2.26 | 0.001 |
| Lateral wall | 0.00 | 0.00 | 4.02 | 0.00 | 0.00 | 0.00 | 0.016 |
|  | LVA (0.5mv in AF) | | | LVA (0.5mv in SR) | | | p value |
| Percentage in LA surface (%) | median | Q1 | Q3 | median | Q1 | Q3 |  |
| Global LA | 25.20 | 16.59 | 50.47 | 12.33 | 4.66 | 29.39 | <0.001 |
| Anterior wall | 9.49 | 2.98 | 18.46 | 3.80 | 0.00 | 6.97 | <0.001 |
| Posterior wall | 7.62 | 3.23 | 11.58 | 3.15 | 0.00 | 7.01 | <0.001 |
| Septal wall | 6.12 | 0.32 | 12.98 | 0.99 | 0.00 | 9.27 | 0.01 |
| Roof wall | 0.83 | 0.00 | 1.61 | 0.00 | 0.00 | 1.39 | 0.118 |
| Inferior wall | 1.81 | 0.54 | 4.54 | 0.43 | 0.00 | 2.26 | 0.001 |
| Lateral wall | 0.00 | 0.00 | 4.02 | 0.00 | 0.00 | 0.00 | 0.023 |

**Supplemental table 1. Spatial distribution and extent of low voltage areas in AF and SR map**

AF. atrial fibrillation; SR. sinus rhythm; LA. left atrium; LVA low voltage area; Q1. 25^th^ percentile; Q3. 75^th^ percentile.

**Supplemental Table 2. Sensitivity analysis**

|  | β Coefficient | p value | Collinearity Statistics | |
| --- | --- | --- | --- | --- |
| Selected variable |  |  | Tolerance | VIF |
| LVA in SR map | 0.08 | 0.004 | 1 | 1 |
| Excluded variable | |  |  |  |
| LVA in AF map | 0.276 | 0.128 | 0.705 | 1.418 |
| LA surface | -0.135 | 0.386 | 0.977 | 1.023 |
| Age | 0.08 | 0.636 | 0.837 | 1.195 |
| Sex | -0.03 | 0.845 | 0.994 | 1.006 |
| Intial Rhythm | 0.187 | 0.23 | 0.962 | 1.04 |
| BMI | -0.026 | 0.866 | 1 | 1 |
| LAD | 0.159 | 0.356 | 0.797 | 1.255 |
| LAV | 0.298 | 0.071 | 0.837 | 1.195 |
| LVEF | -0.16 | 0.301 | 0.988 | 1.012 |
| CAD | 0.277 | 0.071 | 0.973 | 1.028 |
| Creatine | -0.187 | 0.251 | 0.89 | 1.124 |
| Diabets | 0.01 | 0.949 | 0.998 | 1.002 |
| Stroke | 0.279 | 0.064 | 1 | 1 |
| Hypertension | 0.17 | 0.269 | 0.996 | 1.004 |

VIF, variance inflation factor; LVA, low voltage area; SR, sinus rhythm; AF, atrial fibrillation; LA, left atrial; BMI, body mass index; LAD, left atrial diameter; LAV, left atrial volume; LVEF, left ventricular ejection fraction; CAD, coronary artery disease.

Supplemental Figure 1


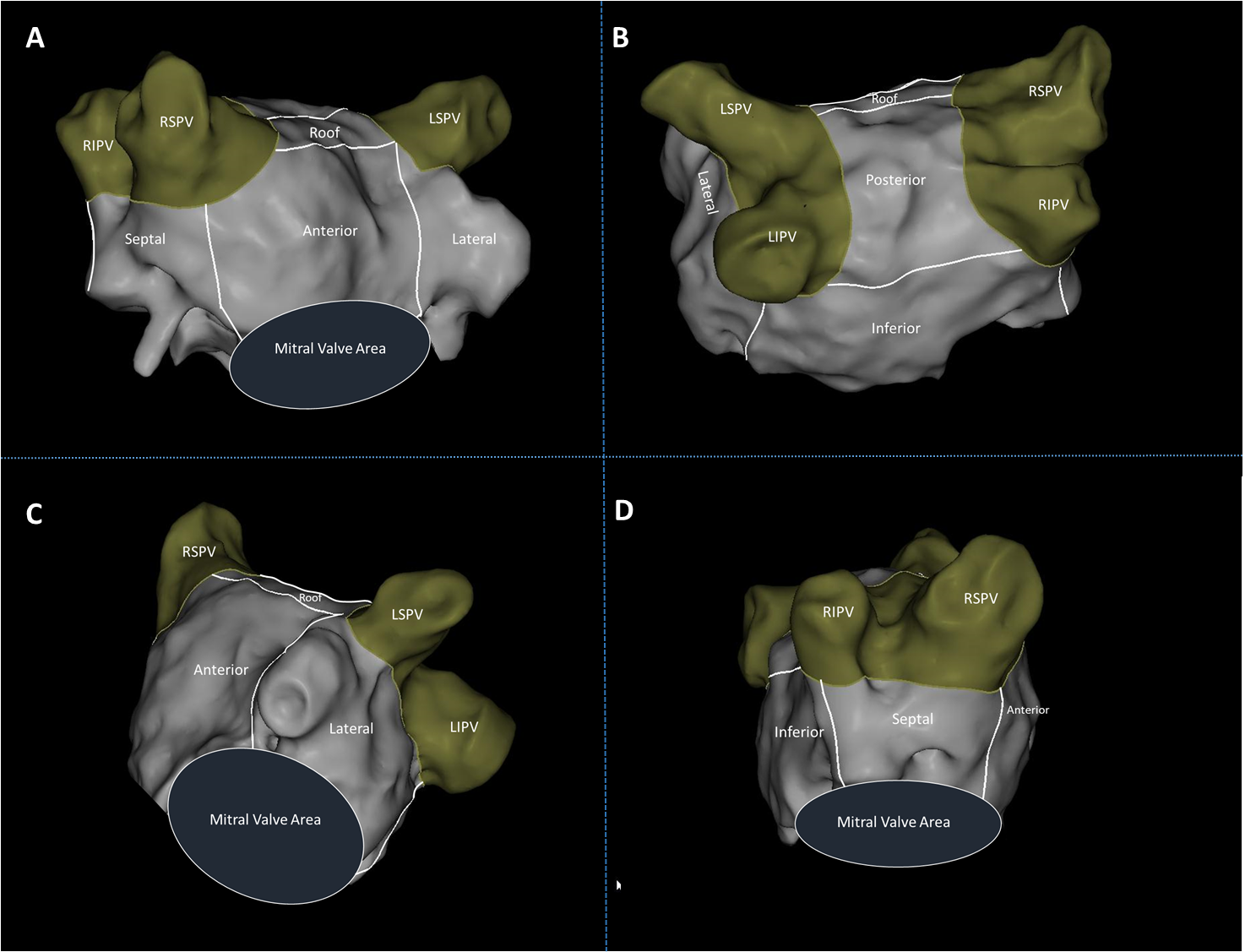


Segmentation of left atrium. Left atrium is divided into six segments in our study including anterior, posterior, septal, roof, inferior and lateral. Panel A, B, C and D illustrate different segments in anterior view, posterior view, lateral view and septal view, respectively.

Supplemental Figure 2


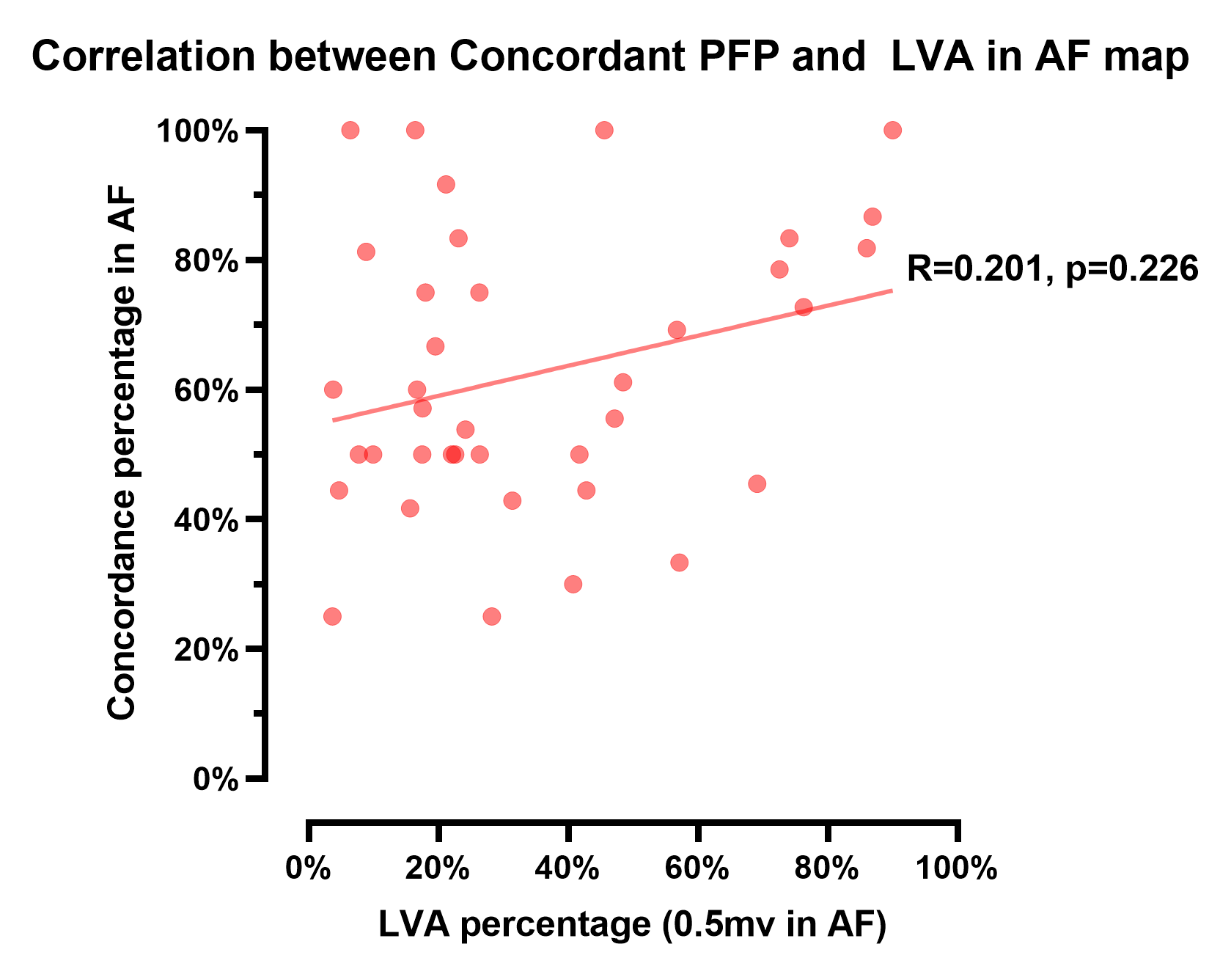


Correlation between percentage of CF in AF map and LVA extent. PFP, prolonged fractionated potential; LVA, low voltage area; CF, concordant fractionation; AF, atrial fibrillation.
